# Utilization of sexual and reproductive health services among young people in refugee settings in Uganda

**DOI:** 10.3389/frph.2023.1077761

**Published:** 2023-02-24

**Authors:** Paul Mukisa Wako Bukuluki, Peter Kisaakye, Symon Peter Wandiembe, Victor Kiwujja, Christine Kajungu, Wilberforce Mugwanya, Shakira Nabakooza, Cyprian Anyii, Fiona Kaikai

**Affiliations:** ^1^ Department of Social Work and Social Administration, Makerere University, Kampala, Uganda; ^2^ Department of Population Studies, Makerere University, Kampala, Uganda; ^3^ Department of Statistical Methods and Actuarial Sciences, Makerere University, Kampala, Uganda; ^4^ Department of Sexual and Reproductive Health and Rights, United Nations Population Fund, Kampala, Uganda

**Keywords:** utilization, sexual and reproductive health services, young people, refugees, Uganda

## Abstract

There is a considerable high level of unmet need for reproductive health services among refugees. Yet, there is limited research about the provision and utilization of sexual and reproductive health (SRH) services among young people in refugee settings. Drawing on a sample of 575 young refugees (15–24 years) from a cross-sectional survey, this study aims to fill this gap by identifying the factors associated with SRH utilization among young people living in refugee settings in Northern Uganda. The utilization of SRH services at the health facilities was significantly different between female and male young people after adjusting for all other variables (aOR = 2.46, 95% CI, 1.58, 3.84). Young people who were not living in a marital union (aOR = 0.38, 95% CI, 0.20, 0.71), or held inequitable gender norms about services (aOR = 0.28, 95% CI, 0.12, 0.66) had about a third of the odds of utilizing SRH services. Young women with comprehensive knowledge about contraception, modern contraceptives, and HIV and STI prevention, had more than twice the odds of utilizing SRH services (aOR = 2.23, 95% CI, 2.67, 6.90). There is need to integrate social norm measurements and social norm change strategies in strategies for promoting utilization of SRH services among refugees in low-income countries especially in Uganda

## Introduction

A significant number of people are said to join humanitarian settings on a yearly basis (1) due to circumstances related to conflict (2) or natural disaster (3). Refugee settings are characterized by a breakdown in provision of services which makes access and utilization of services difficult (4). Yet, utilization of sexual and reproductive health (SRH) services is critical to achieving better wellbeing of individuals (5). Young people are often the most affected negatively regarding access to SRH utilization (6).

Poor SRH service utilization is often associated with refugee settings due to inadequate health facilities and service personnel (7), limited supplies (8), limited funding (4), poor policies (9) and the sensitivity associated with promoting SRH services (10), poor quality of SRH services (8), poor attitudes of health care providers towards young people (11), provider bias (12). Other reasons include health concerns (11), community opposition (13), religious beliefs (14), limited agency to make reproductive health decisions (15), cultural factors (16) or limited self-efficacy (17). These reasons render young people to be neglected (18) or underserved (1). Moreover, the poor state of the health facilities in developing countries is further strained by the refugee crisis, that creates an unmet need for for reproductive health services (19).

Uganda hosts the largest number of refugees in Africa, with a progressive refugee policy that permits refugees to access health care services (20)—providing a better ground to explore SRH utilization (21). Previous research carried out in a refugee setting in Uganda observed that engaging in sexual behavior was more prevalent among out of school adolescents and older adolescents (16–18 years)—which conditions are common in refugee settings in Uganda (22). Refugees like any other person have a right to SRH services, although the capacity to provide SRH services may be limited (23). Previous research observed that unmet need for reproductive health services was higher among refugee settlements in Northern Uganda—where data used in this study come from—compared to the national average (23).

There is limited research about the provision and utilization of SRH services among young people in refugee settings (8), resulting in a knowledge gap regarding the factors associated with SRH utilization among young people living in refugee settings. Moreover, there is limited information regarding the strategies to increases SRH utilization (24). The current study aims to fill this gap by identifying the factors associated with SRH utilization among young people living in refugee settings in Northern Uganda. We focus on refugee settings given the increased vulnerability to poor SRH service utilization among young people in refugee settings (6). Young people with limited access to SRH tend to risk contracting sexually transmitted infections (STIs) (12), experiencing pregnancy related complications (1), unwanted or mistimed pregnancy (19), limiting education attainment (25), increase maternal morbidity (26). Yet, efforts to increase access and utilization of SRH services in refugee settings remain low (17). The analyses from the current study can help in designing strategies aimed at increasing SRH utilization in refugee settings.

## Data and methods

### Source of data and study setting

The current analysis is based on secondary data collected for the baseline study of knowledge, attitudes and practices (KAP) of potential beneficiaries of United Nations Population Fund (UNFPA)'s supported program on Advancing Sexual Reproductive Health and Rights (ANSWER) in Northern Uganda. The survey was conducted in September 2021 among a random sample of 6,056 young people (15–24 years) of which 575 were refugees. The survey was household based using a stratified two-stage cluster design with stratification on districts and urban-rural residence. In the first stage, a probability proportional to the size sample of villages was taken from each stratum. In the second stage, a systematic sample of households with young people (15–24 years) was taken. A response rate of 98% was achieved in the survey.

### Measurement of variables

The dependent variable was whether or not a respondent accessed SRH services or information at the health facilities in the past 12 months. The services included contraceptives and counseling about contraceptives, pregnancy testing, pregnancy termination or post-abortion care, potential factors associated with access and utilization.

The potential factors associated with access and use of SRH services available in the data included socio-demographics, current schooling status [categorized as currently a learner at school (in-school) or out of school], and ever had sex status. Potential factors constructed through alpha factoring, summating or principal component analysis (pca) as scores included household assets index, knowledge about HIV/STI prevention and treatment, comprehensive knowledge of SRH issues (pregnancy prevention, knowledge of contraceptives, prevention of HIV/STI), gender norms score, and community negative perceptions, and life skills. All scores were categorized into binary variables.

*Household asset index* as a proxy measure for the economic wellbeing of a household was constructed through Principal Component Analysis model of household owned domestic items (radio, television set, sofa sets, mattress, solar/electricity for lighting, access to running in the house or yard), transport assets (bicycle, motorcycle, car), and productive assets (computer, mobile phones) and has an income generating activity. Generally, based on the pca scores, households were classified as having high household assets index if they had at least 6 of these 12 items assessed.

*Knowledge of preventing HIV/AIDS and STIs*, and their treatment was obtained from alpha factoring of six items. Respondents were asked to affirm the following: (a) having and being faithful to only one sexual partner is an effective way of preventing HIV, (b) a person can reduce their chances/risk of getting HIV by not having sex, (c) a person can reduce their chances/risk of getting HIV by using condoms when having sex, (d) the HIV/AIDS virus can be transmitted by mosquito bites, (e) the HIV/AIDS virus can be transmitted by supernatural means, (f) a person can become infected by sharing food with a person who has the AIDS virus, (g) a girl or boy cannot get HIV the first time she/he has sexual intercourse, and in addition to knowledge at least two other STIs in addition to HIV/AIDS, and source of their treatment.

*Comprehensive knowledge on SHR* issues was assessed on prevention of pregnancy, knowledge of contraceptives, their use and sources and knowledge of prevention of HIV and STIs, including treatment. A young person was classified to have good comprehensive knowledge if she or he had correct information on at least 80% of the issues or items assessed.

*The score for gender and social norms* was computed from the responses to the following questions, with Likert scale options: (a) boys and girls have equal abilities, (b) Giving a bath and feeding kids are the mother's responsibility, (c) Woman's role is taking care of her home and family, (d) a man should have the final word about decisions in the home, (d) preventing pregnancy is a woman's responsibility, (a) Young people like you/me should not be allowed to use contraceptive services; (b) It is wrong for young girls who are sexually active to use contraceptives, (c) Women who use contraception may become promiscuous.

*Community negative perception* about young people accessing contraceptives and contraceptive information was constructed from affirmative responses (on a Likert scale—very common and common options) of at least two of the following: (a) belief that exposing adolescents to information about sexual health encourages them to start sex, (b) stigmatization of unmarried girls 15–19 years using contraceptives, (c) belief that adolescent girls and young women who carry condoms are promiscuous and cannot be trusted, and (f) belief that girls who use contraceptives are promiscuous.

*Life skills* score for self-efficacy to avoid risky sex, including using a condom was measured by asking the respondents to affirm to the following statements: (a) I am confident I can get the person with whom I have sex to use a condom, even if he/she doesn't want me to use a condom, (b) I am confident If my partner and I do not have a condom, I can say no to sex, (e) I make smart decisions to avoid unsafe sex.

### Data analysis

Data analysis was performed in Stata software Version 15 (27). Descriptive statistics included frequencies and percentages. A binary logistic regression model was fitted at both the bivariate and multivariable analysis levels to identify the factors that were independently associated with accessing SRH services and information in the past 12 months, preceding the survey. Only factors with likelihood ratio test (LRT) *p*-value of less than 0.25 at bivariate level were included in a multivariate model. Age group and current schooling status were included in the multivariable models as apriori factors. Only one variable of a pair with a high correlation of more than 0.35 or odd ratio of association of more than 1.5 was included in the multivariable model. The models were fitted using survey suite of commands in Stata to accounting for the complex sample survey design. Hosmer-Lemeshow goodness-of-fit test was conducted for all the multivariable logistic regression models.

## Results

### Sample characteristics

Of the 575 young refugee people that participated in the survey, over 95% were of South Sudanese origin and a few from Somalia and Democratic Republic of Congo; 303 were male and 272 were female. Of these, 47.5% (49.4% of the males and 45.4% of the females) had ever had sex (Table 1). Forty-one percent were aged 15–17 years and 30.1% (28.1% of the males and 32.4% of the females) were living in a marital union. Further, 56.4% were school-going children, and less than 20% came from households that had at least 7 of the 14 household items assessed.

**Table 1:** Sample characteristics.

	Male		Female		All	
	*n*	%	*n*	%	*n*	%
**Age group**
15–17 years	73	37.1	90	45.3	163	41.0
18–20	130	34.0	88	23.9	218	29.2
21–24	100	28.9	94	30.8	194	29.8
**Current Marital status**
Married/living with a partner	76	28.1	85	32.4	161	30.1
Not in union/Single	227	71.9	187	67.6	414	69.9
**Highest level of education**
None	35	12.2	22	9.2	57	10.8
Primary	144	50.9	177	70.5	321	60.3
Secondary	97	30.5	64	19.0	161	25.0
TVET	27	6.3	9	1.3	36	3.9
**School Status**
In-school	129	56.9	109	55.8	238	56.4
Out of school	174	43.1	163	44.2	337	43.6
Listened to information about SRH on media in past 12 months	303	21.2	272	22.5	575	21.8
**Household Asset Index**
Low	243	82.4	232	85.5	475	83.9
Moderate	60	17.6	40	14.5	100	16.1
**Religion**
Roman Catholic	122	38.3	106	39.3	228	38.8
Anglican/protestant	173	59.8	157	57.5	330	58.7
Moslem	8	2.0	9	3.2	17	2.6
**Disability status**
No disability	262	85.2	234	86.4	496	85.8
Has disability	41	14.8	38	13.6	79	14.2
With good knowledge of HIV and STI prevention	202	68.0	178	66.6	380	67.3
With Life skills—self efficacy	303	82.2	272	82.2	575	82.0
Comprehensive knowledge of SRH issues	303	29.1	272	33.9	575	31.4
Believes the community has negative attitudes toward young people accessing SRH services	303	70.8	272	54.3	575	62.9
Ever had sex	303	49.4	272	45.4	575	47.5
Used SRH services at a health facility in the past 12 month	303	14.0	272	25.2	575	19.3
Sought SRH information in last 12 months	303	27.0	272	21.9	575	24.6

The percentage of the respondents with comprehensive knowledge of pregnancy and contraceptives, and HIV and STI prevention was only 31.4% (29.1% of the males and 33.9% of the females). Further, about three in five respondents had a perception that their communities have negative attitudes toward unmarried young people accessing SRH services. Nonetheless, at least 80% of the young people expressed self-efficacy in avoiding or managing risky behaviors.

### Utilization of SRH services and associated factors

The proportion of young people who had accessed SRH services at the health facilities in the past 12 months preceding the survey was 19.3%; 14.0% among the males and 25.2% among the females (Table 1). In addition, one in four young people reported to have sought SRH information within the past 12 months. The services accessed included: modern contraceptives (reported by 19.6% of respondents), STI screening and treatment (14.2%), pregnancy test or termination (19.8%), antenatal care (15.0%), labor and delivery (4.5%), and HIV testing (23.1%).

At univariate analysis, the key factors associated with young people visiting the health facility in the past 12 months for SRH services included being married, being out of school, ever had sex, being exposed to SRH information on media, and having perceptions that the community has negative attitudes toward unmarried young people utilizing SRH services (Table 2).

**Table 2:** Percentage of young people who visited the health facility for SRH services in the past 12 months and associated factors.

	Male respondents				Female respondents			
	No.	%	Crude OR (95% CI)	*p-*value	No.	%	Crude OR (95%CI)	*p*-value
**Age group**
15–17 years	73	4.0	1		90	8.6	1	
18–20	130	14.1	3.96 (1.26, 12.41)	0.018	88	31.9	4.97 (2.5, 9.88)	0.000
21–24	100	26.7	8.78 (3.14, 24.52)	0.000	94	44.4	8.51 (4.25, 17.03)	0.000
**Current Marital status**
Married/living with a partner	76	34.7	1.00		85	49.6	1.00	
Not in union/Single	227	5.9	0.12 (0.06, 0.24)	0.000	187	13.5	0.16 (0.08, 0.32)	0.000
**Highest level of education**
None	35	10.7	1.00		22	40.7	1.00	
Primary	144	16.7	1.67 (0.62, 4.45)	0.308	177	25.6	0.5 (0.27, 0.92)	0.027
Secondary	97	12.0	1.14 (0.4, 3.24)	0.808	64	16.7	0.29 (0.1, 0.81)	0.018
TVET	27	8.3	0.75 (0.06, 9.14)	0.825	9	20.1	0.37 (0.1, 1.32)	0.125
**Listened to information about SRH on media in past 12 months**
No	232	11.1	1.00		209	23.9	1.00	
Yes	71	24.8	2.64 (1.58, 4.4)	0.000	63	29.6	1.34 (0.72, 2.47)	0.356
**Household Asset Index**
Low	243	11.7	1.00		232	26.1	1.00	
Moderate	60	24.9	2.5 (1.33, 4.7)	0.004	40	20.1	0.72 (0.25, 2.01)	0.526
**School Status**
In-school	129	4.8	1.00		109	12.6	1.00	
Out of school	174	26.2	7.1 (3.01, 16.73)	0.000	163	41.1	4.85 (2.29, 10.27)	0.000
**Ever had sex**
No	148	3.4	1.00		135	11.3	1.00	
Yes	155	24.8	9.24 (3.64, 23.48)	0.000	137	42.0	5.68 (2.44, 13.26)	0.000
**Religion**
Roman Catholic	122	9.0	1.00		106	24.0	1.00	
Anglican/protestant	173	16.8	2.03 (1.21, 3.4)	0.007	157	26.9	1.17 (0.66, 2.05)	0.596
Moslem	8	26.5	3.63 (0.78, 16.83)	0.099	9	9.0	0.31 (0.04, 2.74)	0.293
**Disability status**
No disability	262	14.1	1.00		234	25.1	1.00	
Has disability	41	13.7	0.969 (0.295, 3.18)	0.960	38	25.8	1.036 (0.35, 3.06)	0.950
**Knowledge of pregnancy prevention, contraceptive methods, HIV and STI prevention**
Limited	212	12.3	1.00		178	16.4	1.00	
Good	91	17.5	1.47 (0.76, 2.86)	0.155	94	42.3	3.73 (1.87, 7.49)	0.000
**Believes the community has negative attitudes toward young people accessing SRH services**
No	90	4.5	1.00		128	21.1	1.00	
Yes	213	18.8	14.03 (5.41, 36.31)	0.000	144	31.3	1.66 (0.66, 3.77)	0.157
**Gender norms score**
Positive	145	18.9	1.00		166	26.4	1.00	
Negative	158	8.8	0.41 (0.20, 0.85)	0.017	106	23.5	0.86 (0.45, 1.64)	0.649

There were also associated factors with SRH service utilization specific to either male or female respondents. Among the males, those with inequitable gender norms, and from poor households were least likely to have visited the health facility for SRH services within the past 12 months. While, among the females, comprehensive knowledge of SRH also positively influenced utilization of SRH services.

### Multivariable analysis of factors associated with the utilization of SRH services by young people (all) at the health facility

The utilization of SRH services at the health facilities was significantly different between female and male young people after adjusting for all other variables (aOR = 2.46, 95% CI, 1.58, 3.84). Results of multivariable analysis of factors associated with male and female young people using SRH services at the health facility within the last 12 months are presented in Table 3.

**Table 3:** Multivariable analysis of factors associated with Use of SRH services at the health facility in the past 12 months by young people (*n* = 575).

	Male respondents			Female respondents		
	adj ORs	pval	(95% Conf. Interval)	adj ORs	pval	(95% Conf. Interval)
**Age group**
15–17 years	1.00			1.00		
18–20	1.12	0.796	(0.49, 2.55)	2.20	0.069	(0.94, 5.16)
21–24	0.96	0.937	(0.39, 2.36)	2.28	0.023	**(1.12, 4.64)**
**Marital status**
Married/living with a partner	1.00			1.00		
Not in union/Single	0.38	0.113	(0.11, 1.26)	0.38	0.046	**(0.15, 0.98)**
**Listened to information about SRH on media in past 12 month**
No	1.00			1.00		
Yes	4.29	0.000	**(2.67, 6.90)**	1.45	0.219	(0.80, 2.64)
**Household Asset Index**
Low	1.00			1.00		
Moderate	1.87	0.209	(0.70, 5.00)	1.86	0.202	(0.72, 4.82)
**School Status**
In-school	1.00			1.00		
Out of school	4.12	0.003	**(1.63, 10.47)**	1.14	0.851	(0.29, 4.41)
**Religion**
Roman Catholic	1.00			1.00		
Anglican/protestant	2.75	0.000	**(1.86, 4.06)**	1.68	0.189	(0.78, 3.63)
Moslem	6.44	0.012	**(1.52, 27.38)**	0.26	0.214	(0.03, 2.16)
**Knowledge of pregnancy prevention, contraceptive methods, HIV/STI prevention**
Limited	1.00			1.00		
Good	0.94	0.882	(0.41, 2.17)	2.37	0.028	**(1.10, 5.09)**
**Gender norms**
Positive	1.00			1.00		
Negative	0.30	0.008	**(0.12, 0.73)**	0.64	0.247	(0.31, 1.36)
**Believes the community has negative perceptions toward young people accessing SRH services**
No	1.00			1.00		
Yes	10.04	0.000	**(3.09, 32.61)**	2.03	0.303	(0.63, 7.50)

Significant values in bold.

Among the male young people in refugee settlements, access and utilization of SRH services was low among individuals who believed in inequitable gender norms and were Roman Catholics. Individuals who believed in inequitable gender norms had about a third of the odds of accessing SRH services at the health facilities as those with equitable gender norms (aOR = 0.30, 95% CI, 0.12, 0.73). The Anglicans and other protestants had about three times the odds of utilizing SRH services (aOR = 2.7, 95% CI, 1.86, 4.06) than the Roman Catholics. Exposure to information about SRH in the past 3 months through media was also associated with increased odds of utilizing SRH services within the past 12 months. Further, individuals who held the perception that the community (including health workers) has negative perceptions about unmarried young people accessing SRH services had more than ten times the odds of utilizing SRH than those without such perceptions (aOR = 10.04, 95% CI, 3.09, 32.61). The association suggests the likelihood of young people experiencing community reactions after they have accessed SRH services.

However, among the female young people, both religion and beliefs inequitable gender norms did not influence the utilization of SRH services at the health facility within the past 12 months. Moreover, unlike boys and young men, girls, and young women with comprehensive knowledge about contraception, modern contraceptives, and HIV and STI prevention, had more than twice the odds of utilizing SRH services as compared to those with limited knowledge (aOR = 2.23, 95% CI, 2.67, 6.90). Whereas the proportion of girls and young women who held perceptions that the community has negative attitudes toward unmarried young people accessing SRH services had twice the odds of utilizing SRH services, this was not significant (2.03, 95% CI, 0.63, 7.50).

### Multivariable analysis of factors associated with utilization of SRH services by sexually active young people

Of the 223 young people who had sex within the past 12 months, 164 had sex more than once but were not on contraceptives. Among these 164, 32.4% (23.6% of males and 41.1% of females) reported having sought SRH services from health facilities within the past 12 months. The key factors independently associated with the utilization of SRH services included being female, married, and with equitable gender norms (Table 3). Table 4 shows that young people who were not living in a marital union had about a third of the odds of married/cohabiting young people utilizing SRH services (aOR = 0.38, 95% CI, 0.20, 0.71). Similarly, young people who hold inequitable gender norms had about a third of the odds of those with equitable gender norms utilizing SRH services (aOR = 0.28, 95% CI, 0.12, 0.66). In addition, young people who had perceptions that the community had negative attitudes toward unmarried young people accessing SRH services had high odds (aOR = 3.23, 95% CI, 1.10, 9.73) of utilizing SRH services.

**Table 4:** Multivariable analysis of factors associated with Use of SRH services at the health facility in the past 12 months by the sexually active young people (*n* = 164).

	*n*	**% who visited the health facility**	adj. ORs	*p*-value	(95% Conf. Interval)
**Sex**
Male	80	23.6	1.00		
Female	84	41.1	2.81	0.009	**(1.3, 6.08)**
Age group					(1.3, 6.08)
15–17 years	25	6.3	1.00		
18–20	74	26.5	2.42	0.198	(0.63, 9.24)
21–24	65	46.4	4.88	0.017	**(1.33, 17.95)**
**Marital status of respondent**
Not in union/Single	94	44.5	1.00		
Married/living with a partner	70	13.2	0.38	0.003	**(0.2, 0.71)**
**Listened to information about SRH on media in past 12 months**
No	139	29.9	1.00		
Yes	25	48.3	2.26	0.233	(0.59, 8.61)
**Household Asset Index**
Low	138	32.7	1.00		
Moderate	26	30.5	0.81	0.692	(0.28, 2.35)
**School Status**
In-school	32	11.7	1.00		
Out of school	132	40.6	1.86	0.406	(0.43, 8.02)
**Religion**
Roman Catholic	66	25.7	1.00		
Anglican/protestant	94	37.7	2.43	0.045	**(1.02, 5.81)**
Moslem	4	25.0	0.62	0.383	(0.21, 1.82)
**Knowledge of pregnancy prevention, contraceptive methods, HIV and STI prevention**
Limited	108	29.9	1.00		
Good	56	37.4	0.80	0.495	(0.42, 1.53)
**Gender norms**
Positive	76	45.0	1.00		
Negative	88	20.7	0.28	0.004	**(0.12, 0.66)**
**Believes the community has negative attitudes toward young people accessing SRH services**
No	48	29.2	1.00		
Yes	116	33.7	3.23	0.038	**(1.07, 9.73)**
**Life skills score**	164	…	1.02	0.852	(0.84, 1.23)

Significant values in bold.

## Discussion

Our results demonstrate that among young people living in refugee settlements, the key factors independently associated with the utilization of SRH services included being female, married, and with equitable gender norms. This further confirms studies that have underscored the importance of gender and social norms in influencing utilization of SRH services among young people in refugee settlements or humanitarian settings (5, 11, 28).

Our results show gender differences in utilization of SRH services at the health facilities female and male young people (refugees). For example, girls and young women with comprehensive knowledge about SRH services were more likely to use SRH services than their male counterparts. This suggests that for boys and young men comprehensive knowledge about SRH is inadequate to facilitate use of SRH services. It confirms previous studies that argue that knowledge alone is inadequate to facilitate behavioral change (1, 11, 22). Inequitable gender norms show significant influence in access and utilization of SRH services at facilities in refugee settlements (11, 29) particularly among male young people. However, among the female young people, inequitable gender norms did not influence the utilization of SRH services at the health facility. This points to the relative strengths of social and gender norms in specific contexts and the need to explore which norms have stronger influence on males and females in specific contexts and SRH services (30, 31). These results emphasize the need for integrating a gender lens in promoting utilization of SRH services among young people in refugee settlements. This confirms findings from other studies that emphasize that intervention package for male and female young people in refugee settlements should take into account their peculiar gender needs and contexts (32) that may influence uptake of SRH services.

Our results show that some social norms had no a strong effect on utilization of SRH services among young people in refugee settlements. For example, individuals who held the perception that the community has negative perceptions about unmarried young people accessing SRH services were more likely to utilize SRH than those without such perceptions. This suggests that young people had self-efficacy to challenge or go against the social/community expectations (injunctive norms) that would affect utilization of SRH services. This is in line with current debates that show that in specific contexts some social norms may not have the functional strengths to influence practices or behaviors (33). This also points to the manifestations of what has been conceptualized as pluralistic ignorance which means that individuals may think that their personal beliefs, ideas or feelings are different from others but their public behavior should be the same.

Our results show that marital status is an important factor in influencing access and utilization of services (13, 34). For example, in our study, young people who were not living in a marital union had about a third of the odds of married/cohabiting young people utilizing SRH services. Therefore, being unmarried influences the kind of barriers or enablers young people in refugee settlements have to navigate in access and utilization of services (13, 35). This further points to the importance of having designing interventions that appreciate the unique or peculiar characteristics and context of married and unmarried young people in refugee settings.

## Conclusion and implications

Taken together, the key factors independently associated with the utilization of SRH services among adolescents living in refugee settlements in Uganda included being female, married, and with equitable gender norms. This further emphasizes the need to integrate social norm measurements and social norm change strategies in strategies for promoting utilization of SRH services among refugees in low-income countries especially in Uganda. These results also point to the need to pay attention to context specificity as well as gender sensitivity in designing and implementing SRH interventions in targeting young people in refugee settlements. There is also need to pay attention during social norm diagnosis and measurement to the relative strengths of some social norms over others in influencing uptake of SRH services. Our results that show differences by marriage and gender in influencing SRH services access and utilization point to the need to continue emphasizing audience segmentation in design and delivery of SRH interventions particularly that have social behavioral change activities to facilitate addressing the unique or peculiar needs as well as barriers and enablers to access and utilization of SRH services by young people in refugee settlements or humanitarian settings.

## Data Availability

The original contributions presented in the study are included in the article/Supplementary Material, further inquiries can be directed to the corresponding author/s.
